# Supplementary material for: The Pseudomonas aeruginosa accessory genome elements influence virulence towards Caenorhabditis elegans
Source: Genome Biol. 2019 Dec 10;20:270. doi: 10.1186/s13059-019-1890-1 (PMC6902481; doi:10.1186/s13059-019-1890-1)
Supplement: Supplementary file 1 — Additional file 1. Supplemental Figures S1 to S8. [file 13059_2019_1890_MOESM1_ESM.pdf]

Additional file 1

Figure S1. Traits of the interaction between *C. elegans* and *P. aeruginosa* isolates.

A) Box-and-whisker plot for viable progeny counts for *P. aeruginosa* z7 and *E. coli* HB101. Adult *C. elegans* hermaphrodites were exposed to the above-mentioned bacterial strains using the same conditions for virulence assay with the exception that no FUDR was added (SK plates, 25ºC). The total progeny of individual worms was manually counted. Comparison of the two conditions was done using the Welch *t*-test (p-value indicated). **B)** Box-and-whisker plot of worm median survival in relationship with strain source (environmental or clinical). p-value is indicated for the Welch *t*-test comparison of virulence (*i.e.* induced worm median survival) between clinical and environmental strains.

Figure S2. Relationship between bacterial growth rate and virulence.

Association between bacterial growth rates in LB medium (μ) and virulence among *P. aeruginosa* strains (median survival in days).

Figure S3. Association between non-coding RNA and previously identified virulence genes and virulence.

A) Association between non-coding RNAs of *P. aeruginosa* and bacterial virulence: (Top panel) Median survival of adult *C. elegans* worms (with 95% confidence interval, C.I.), similar to Figure 2B. (Bottom left panel): gene presence/absence matrix for non-coding RNAs. Presence is indicated with black squares and absence with white squares. Non-coding RNAs (rows) are aligned with the corresponding MW and LR p-values (bottom right panel), shown as $|\log_{10}(\text{pval})|$. Rows are ordered from association with high virulence to association with low virulence. **B**) Distribution and association of previously identified virulence genes. (Top panel) Median survival of adult *C. elegans* worms (with 95% confidence interval, C.I.) exposed to the studied collection of *P. aeruginosa* strains. The strains are ordered from high to low virulence (left to right) and aligned with the matrix below. (Bottom left) gene presence/absence matrix for known virulence genes. Gene presence is indicated with black squares and absence with white squares. Genes (rows) are aligned with the corresponding p-values. (Bottom right) Association statistics (p-value of MW and LR tests) for the genes (shown as |log10(p-value)|). Rows are ordered from association with high virulence to association with low virulence.

Figure S4. Bacterial virulence upon loss of mexZ gene.

Survival curves (left panel) and median survival (right panel, with 95% confidence interval 'C.I.') of adult *pmk-1(lf) C. elegans* worms exposed to wild-type and *ΔmexZ* strains of *P. aeruginosa* z8. Pairwise comparison of the survival curves between the two strains was done using the logrank test. The test p-value is indicated in the curve legend.

Figure S5. Relationship between CRISPR-Cas systems and virulence.

(**A**) Box-and-whisker plot of the relationship between CRISPR-Cas subtypes and virulence. Strains are categorized by their combination of CRISPR-Cas subtypes. Strains with type CRISPR-Cas I-C systems have significantly lower virulence than their complementary strain set (Welch t-test, p-value = 0.03). (**B**) K-M Survival curves of adult *C. elegans* worms exposed to the studied collection of 52 *P. aeruginosa* strains partitioned according to presence (in cyan color) or absence (in red color) of host CRISPR-Cas

systems. Survival data for the pooled strains were aggregated as described in Materials and Methods. The p-value of a long-rank test between the two subgroups is indicated. (**C**-**D**) Survival curves (left panels) and median survival (right panels, with 95% confidence interval ‘C.I.’) of adult *C. elegans* worms exposed to strains of *P. aeruginosa*. (**C**) Virulence of PA14 wildtype and PA14 with deletion of the type I-F Cas genes (ΔCas). (**D**) Virulence of PAO1 wildtype; PAO1 with plasmid expressing the type I-F Cas genes (pCas^{+}); PAO1 with control plasmid (ctl plasmid). Pairwise comparison of the survival curves was done using the logrank test. The p-values are indicated in the respective legend.

Figure S6. Relationship between Restriction-Modification (RM) systems and virulence.

A-**E)** Box-and-whisker plots of worm median survival (virulence) in relationship with the abundance and type of RM systems. **A**) The total number of RM systems per strain is displayed. **B**-**E**) The number of RM systems per strain is displayed separately for type I (**B**), II (**C**), III (**D**) and IV (**E**) systems. Correlation values are indicated in all graphs (✈, Spearman rank correlation) except (**D**-**E**) where the RM frequency precludes the confident assesment of association. The median virulence of the complete set of strains displayed on each graph is indicated with the dashed horizontal line.

Figure S7. Relationship between recently described defense systems and virulence.

(**A**-**E**) Box-and-whisker plots of worm median survival in relationship with novel defense system abundance and types. The presence/absence of six novel systems in relationship with median worm survival, displayed separately for wadjet (**A**), gabija (**B**), druantia (**C**), shedu (**D**) and zorya (**E**) systems. **F**) The total number of novel systems per strain is displayed. **G**) The presence/absence of novel defense systems is displayed. In all graphs, no difference in virulence compared to their complementary strain sets is observed (Welch t-test, all p-values > 0.05). The median virulence of the complete set of strains displayed on each graph is indicated with the dashed horizontal line.

Figure S8. Comparison between two methods to determine median survival.

Median survival of adult C. elegans worms exposed to four P. aeruginosa strains (confidence interval, C.I.) scored with two distinct methods. The methods to obtain the median survival estimates are: semi-automated scanning procedure (referred to as ‘machine’); manual scoring with a pick (referred to as ‘manual’).

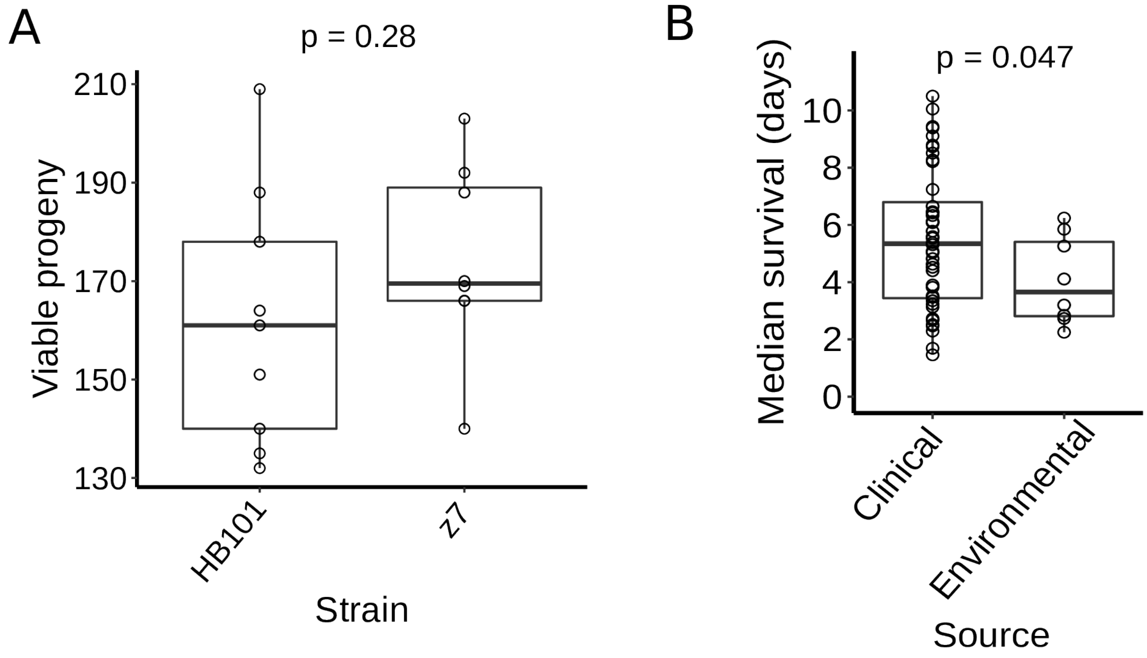
A
p = 0.28
Viable progeny
210
190
170
150
130
HB101
z7
Strain
B
p = 0.047
Median survival (days)
10
8
6
4
2
0
Clinical
Environmental
Source

Figure S1

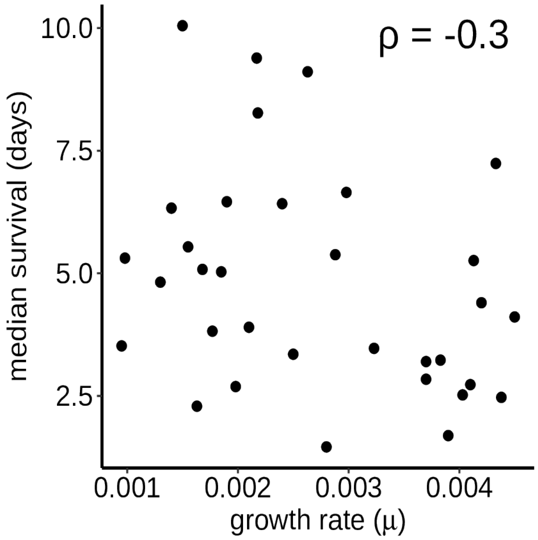
ρ = -0.3
median survival (days)
10.0
7.5
5.0
2.5
0.001
0.002
0.003
0.004
growth rate (μ)

Figure S2

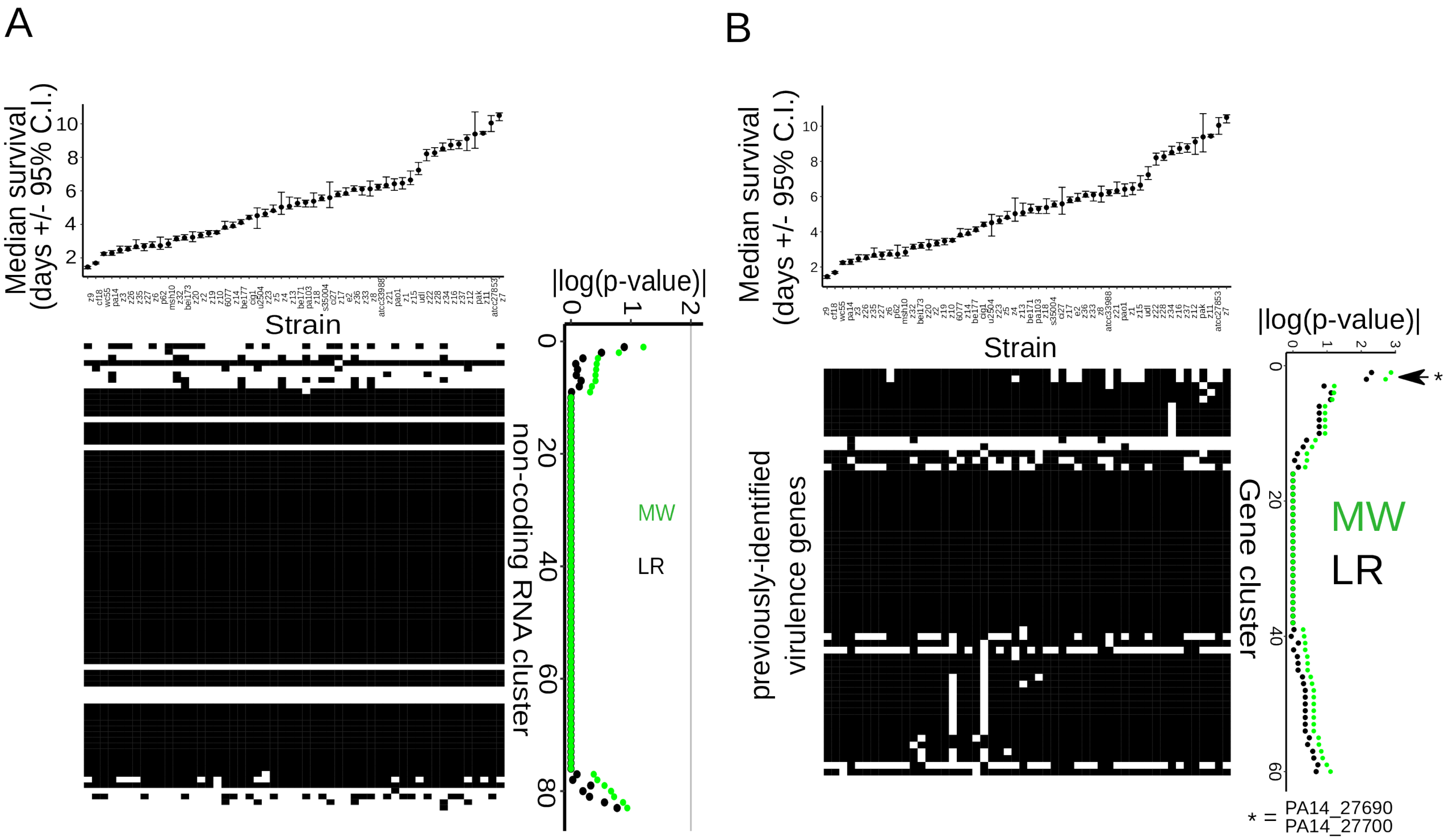
A
B
Median survival (days +/- 95% C.I.)
Strain
|log(p-value)|
non-coding RNA cluster
MW
LR
previously-identified virulence genes
Gene cluster
* = PA14_27690 PA14_27700

Figure S3

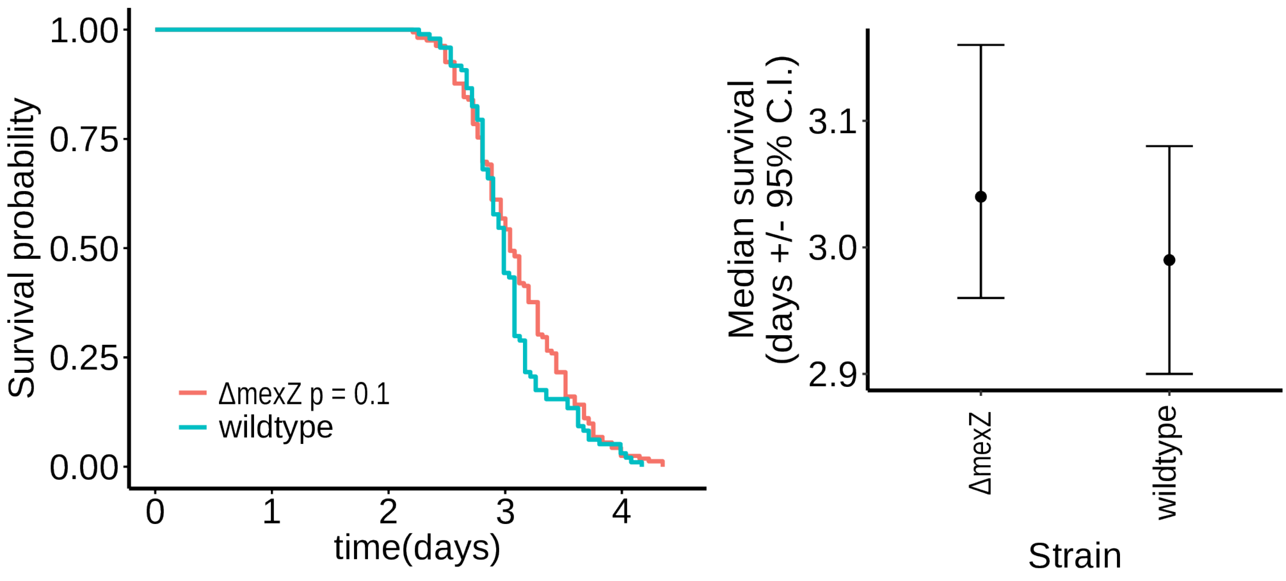

Survival probability
1.00
0.75
0.50
0.25
0.00
ΔmexZ p = 0.1
wildtype
0
1
2
3
4
time(days)
Median survival
(days +/- 95% C.I.)
3.1
3.0
2.9
ΔmexZ
wildtype
Strain

Figure S4

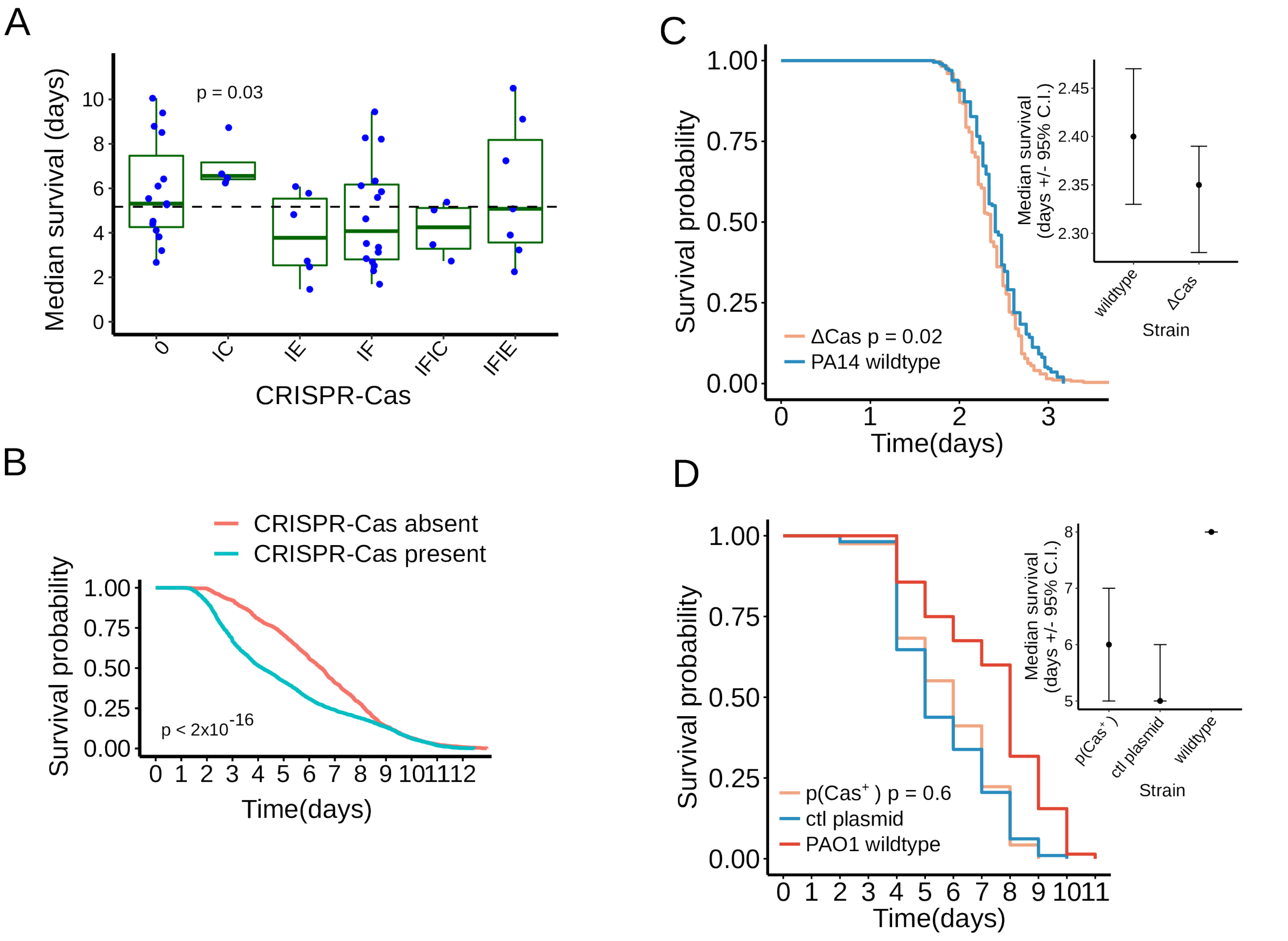

A
Median survival (days)
p = 0.03
CRISPR-Cas
0
IC
IE
IF
IFIC
IFIE
B
CRISPR-Cas absent
CRISPR-Cas present
Survival probability
p < 2x10-16
Time(days)
C
Survival probability
ΔCas p = 0.02
PA14 wildtype
Time(days)
Median survival (days +/- 95% C.I.)
wildtype
ΔCas
Strain
D
Survival probability
p(Cas+) p = 0.6
ctl plasmid
PAO1 wildtype
Time(days)
Median survival (days +/- 95% C.I.)
p(Cas+)
ctl plasmid
wildtype
Strain

Figure S5

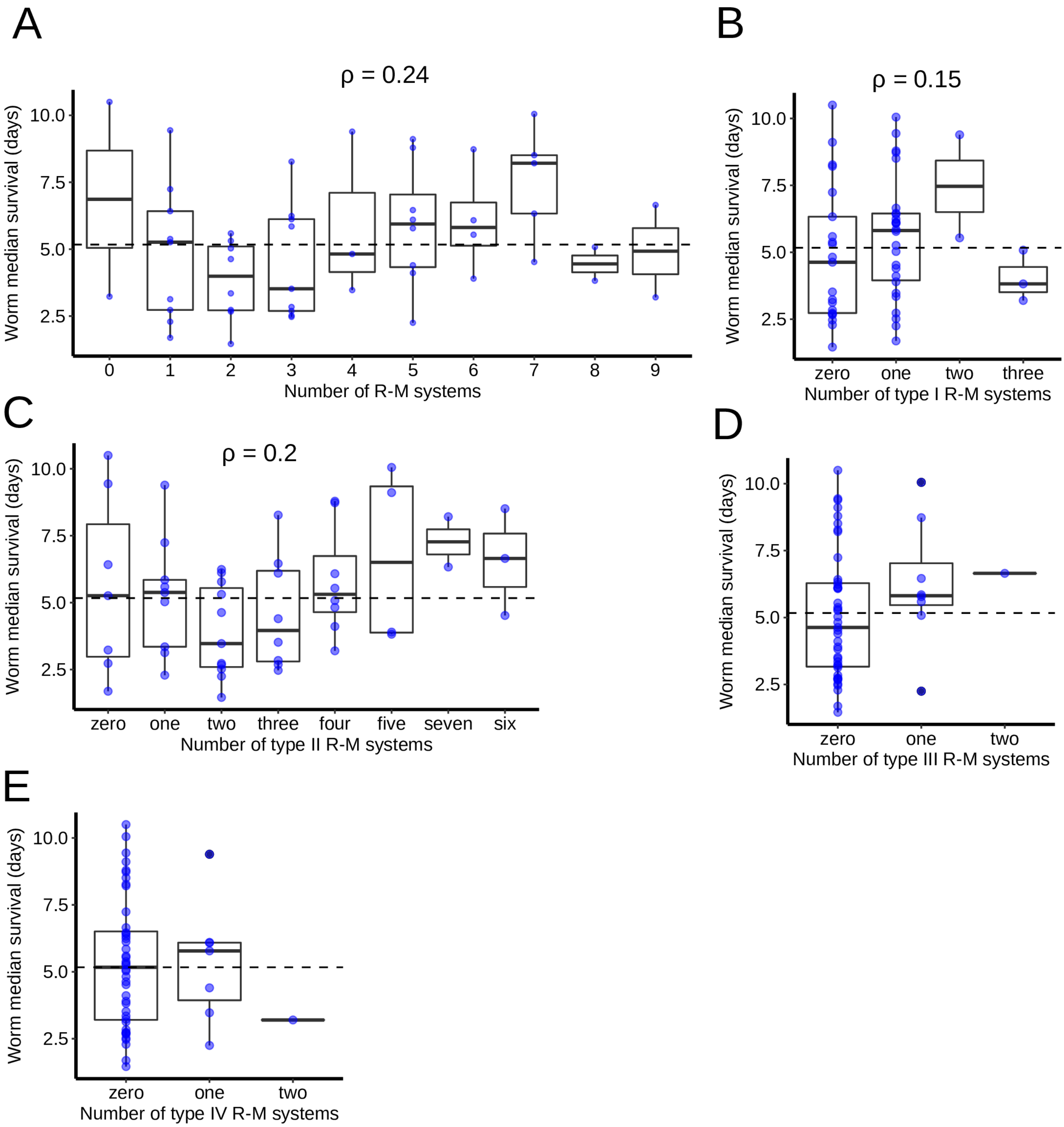
A
ρ = 0.24
Worm median survival (days)
10.0
7.5
5.0
2.5
0
1
2
3
4
5
6
7
8
9
Number of R-M systems
B
ρ = 0.15
Worm median survival (days)
zero
one
two
three
Number of type I R-M systems
C
ρ = 0.2
Worm median survival (days)
zero
one
two
three
four
five
seven
six
Number of type II R-M systems
D
Worm median survival (days)
zero
one
two
Number of type III R-M systems
E
Worm median survival (days)
zero
one
two
Number of type IV R-M systems

Figure S6

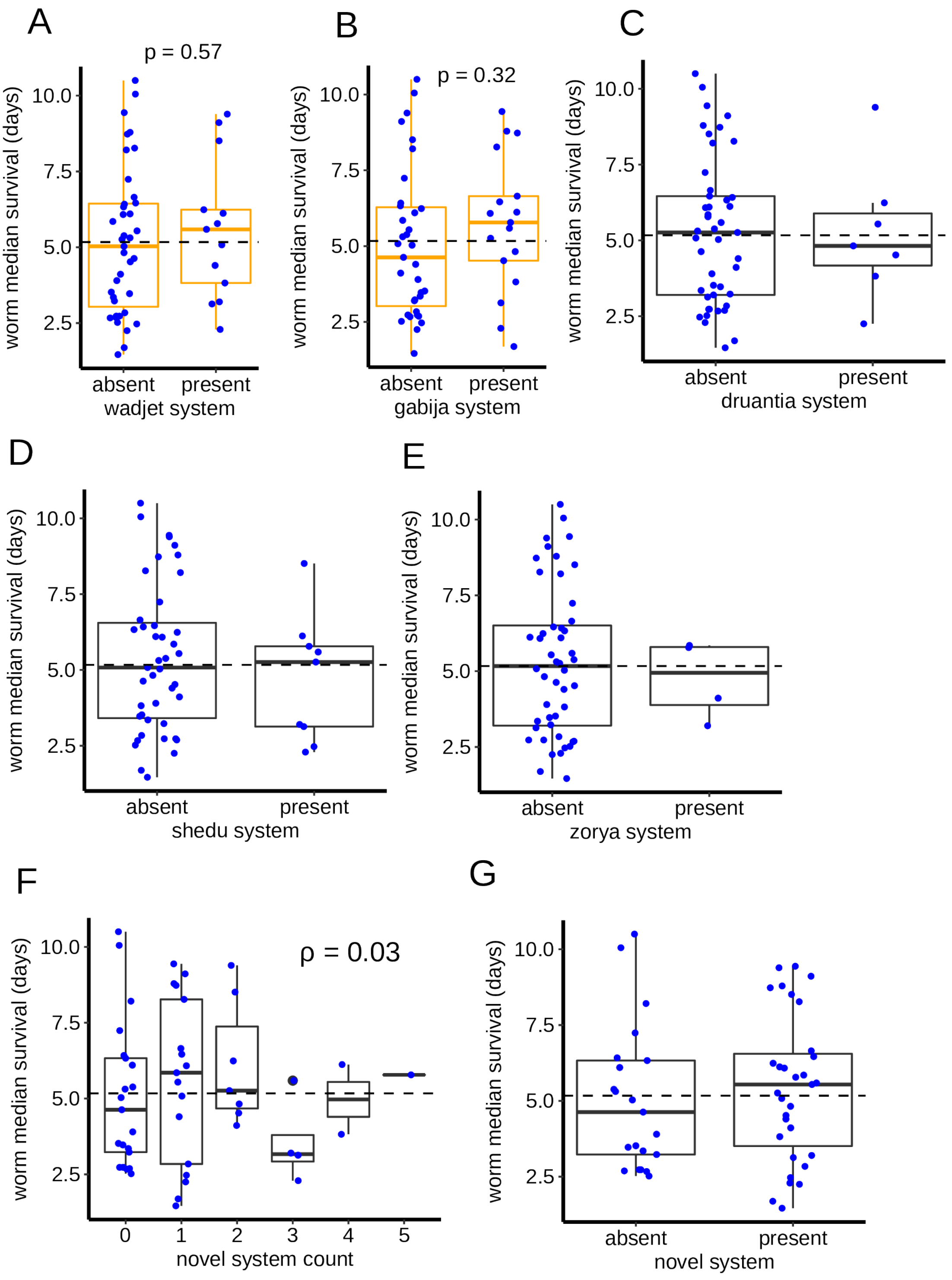
A
p = 0.57
worm median survival (days)
10.0
7.5
5.0
2.5
absent
present
wadjet system
B
p = 0.32
worm median survival (days)
absent
present
gabija system
C
worm median survival (days)
absent
present
druantia system
D
worm median survival (days)
absent
present
shedu system
E
worm median survival (days)
absent
present
zorya system
F
ρ = 0.03
worm median survival (days)
0
1
2
3
4
5
novel system count
G
worm median survival (days)
absent
present
novel system

Figure S7

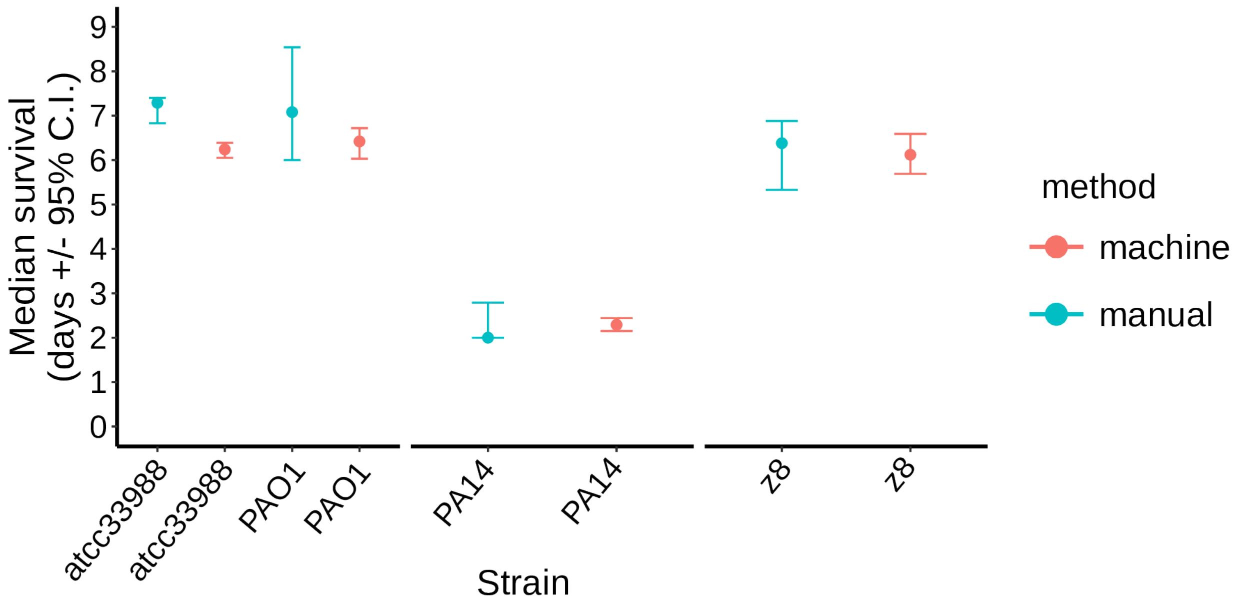

Median survival
(days +/- 95% C.I.)
0
1
2
3
4
5
6
7
8
9
atcc33988
atcc33988
PAO1
PAO1
PA14
PA14
z8
z8
Strain
method
machine
manual

Figure S8
