# Supplementary material for: The Pseudomonas aeruginosa accessory genome elements influence virulence towards Caenorhabditis elegans
Source: Genome Biol. 2019 Dec 10;20:270. doi: 10.1186/s13059-019-1890-1 (PMC6902481; doi:10.1186/s13059-019-1890-1)
Supplement: Supplementary file 11 — Additional file 11. Review history. [file 13059_2019_1890_MOESM11_ESM.docx]

**1^st^ round**

**Reviewer 1**

In this manuscript, the authors assess the genetic and genomic bases of P. aeruginosa virulence against C. elegans hosts. They test a panel of P. aeruginosa isolates (some clinical, some environmental) for host mortality and host fecundity. Then, they use genetic association and genetics methodology to determine the portions of the pathogen genome associated with virulence in C. elegans. Overall, their findings support several previously identified pathogen loci linked to virulence and they also find novel loci associated with virulence. Of particular interest are teg blocks that are associated with decreases in pathogen virulence.

Overall, the findings are a significant advance in host-pathogen interactions and the experimental work is quite thorough. However, I have several major comments about the manuscript writing and statistical analyses.

1. The introduction does not motivate the study effectively. The authors convey in the introduction that they "addressed the sources and genomic correlates of variability in the virulence of distinct P. aeruginosa strains towards C. elegans" (page 2 lines 11-12). It is not clear to me from the current introduction why variability, or the source of variability, in P. aeruginosa virulence towards C. elegans is worth pursuing. What do you hope to learn from this pursuit and why? The first several paragraphs of the introduction don't seem to address this question. Given that many different strains of P. aeruginosa likely interact with many different strains of C. elegans in nature (and your work explores on the pathogen side of this genetic variation...as one host genotype is primarily used throughout), I recommend an introduction that is more focused on variability in P. aeruginosa. I believe this will be more effective at framing the idea that bacterial immune systems may play a role in moderating virulence.

2. Host-pathogen interactions can vary broadly under different environmental conditions (see Leung, J. M., S. A. Budischak, H. Chung The, C. Hansen, R. Bowcutt, R. Neill, M. Shellman, P. n. Loke, and A. L. Graham. 2018. Rapid environmental effects on gut nematode susceptibility in rewilded mice. PLOS Biology 16:e2004108). This should be taken into account when assessing the loci underlying virulence in the lab. This does not diminish your results in any way, but it is important to recognize that virulence may be environmentally mediated and thus the loci underlying virulence may be conditional.

3. It does not seem statistically appropriate to use Spearman rank correlations on data sets in which some data categories have very few data points. For example Supplemental Figure 6E has one Type IV R-M system with only a single data point. I recognize that this analysis and several others like it find no significant association between variables analyzed, so perhaps this is a moot point. However, it would seem more appropriate to exclude these small data sets from the analysis.

4. I generally understand the hypotheses the authors present in Figure 9, the model linking CRISPR and virulence. However, the model figure itself (Figure 9), does not make intuitive sense to me.

5. Rather than ending the conclusion with potential missed loci, I recommend ending by expanding on the CRISPR and virulence association and what it might mean for bacterial genome evolution as a whole. Essentially put this result in a broader context.

**Reviewer 2**

This is a comparative genomic analysis of Pseudomonas aeruginosa isolates that differed in their virulence towards Caenorhabditis elegans. A number of accessory genes were identified that were associated with high-virulence or low-virulence isolates. Mutants containing disruptions of several of these genes and over-expression constructs were generated to measure the contribution of these gene to virulence towards C. elegans. Some of these showed the expected changes while others did not. Further analyses provided evidence supporting the model that active CRISPR-CAS systems are associated with high-virulence strains, and that some CRISP-CAS systems have targeted accessory genomic elements associated with low-virulence. The manuscript is in general well written and provides new information that the readership of Genome Biology will find interesting. There are, however, several issues that must be addressed before the strength of the conclusions can be assessed.

Major points:

1. More information on the C. elegans virulence assays should be provided. In particular, approximately how many worms were tested for each isolate? Are the life-span values shown in Fig. 2 derived from a single plate of worms or from multiple plates? If multiple, how many?

2. It appears that no correction for multiple comparisons was made to the statistics describing the gene association studies. This should be done. It is anticipated that some of the HVA and LVA genes will no longer be statistically significantly associated with virulence phenotypes after this correction is made, and that this explains why several of the genes were thought to be associated with virulence but did not have a phenotype when deleted or over-expressed. If this is indeed the case, the discussion should be revised accordingly.

3. LVA genes might also decrease virulence if their insertion in the chromosome disrupted a gene that enhanced virulence. For LVA genes that did not have the expected phenotypes, this possibility should be examined (i.e. their genomic context should be examined to see whether they appear to have inserted within genes).

4. The phylogenetic trees should be corrected to remove the effects of recombination regions to more accurately show the phylogenetic relationships between the isolates. In particular, TreeWas is meant to be used with trees for which recombination sequences have been removed.

5. page 19, lines 34-36: The reason stated for excluding type I-C CRISPR-Cas systems in this analysis is not compelling. One would expect that these ICE-encoded CRISPR-Cas systems would benefit the ICE by acting as standard immunity systems to prevent the death of the host bacterium and therefore should also prevent the uptake of LVA genes. Either a better rationale for excluding the type I-C systems should be provided or these systems should be included in the overall analysis.

6. Supplemental Figure 5, panel D. Could the authors speculate on why the survival curves differ so much between the PAO1 wildtype and the PAO1 with the empty plasmid?

7. Generation of z8 strains: Either more details should be provided or a reference should be added to help the reader who is unfamiliar with this strategy.

Minor points:

1. The Figure 3 panels are labeled incorrectly according to the Fig. 3 legend.

2. It is appreciated that repeatedly showing the "Median survival vs. Strain" curve helps the reader interpret the gene matrices in the different figures. However, since the repetition of this curve is shown to inform the axes of these matrices and not to provide new information, it should be included in the matrix figure panels and not shown as separate panels. For example, in figure 7 panel A and panel B should be combined into a single panel. Panel A provides no new information and should therefore not be a separate panel.

3. page 6, line 53: Should "mean" be "median"?

4. page 21, line 32: A parenthesis is missing after "Figure 7."

**Reviewer 3**

The manuscript from Vasquez-Rifo et al. describes the use of a GWAS approach to identify genes of Pseudomonas aeruginosa that are correlated with higher and lower virulence in a slow killing model with C. elegans. Overall, there is quite a bit of work that went into this manuscript, and the results are solid.

My one main critique is that this manuscript is quite dense to read. I wonder if there is a way to parse the information more clearly and to *just* focus on the main results within the main body of the manuscript. As such there are a variety of tests and associations and it's a significant effort to wade through everything to digest the results that are worth digesting. I'm a fan of including as much information as possible, usually, but there is a lot in this manuscript and I think it really help the readers to move the negative results (other immune systems, some of the low virulence genes, etc...) to a supplemental document.

Other than this main critique on clarity, I really only have a few minor points that the authors should consider before publication:

Page 2, L31 and throughout: you keep using the word "indirect" here. I think it would be good, at least once, to mention that these are truly correlations and that doesn't imply causation. Using the word "indirect" kind of glosses over this point, the association is real, it's just not necessarily a causative association.

Page 2, Line 58 (and a couple of other places, page 3 L6 etc...): there are statements included like "and do not associate with any clade in particular". Use of this statement is inherently subjective. It may look like there are no associations to the eye, but there might be some there still. Would be good to include some data showing lack of bias in the sampling of strains or the phylogenetic clustering of strains to back up statements like this.

Figure 3: I think the labels are off here as the legend specifies A-D but the labels go from A-E.

Page 13, L29-33: I think there's a typo here. I think the second use of qsrO should actually read ghlO?..."In contrast, no difference in virulence was observed for strains overexpressing the qsrO (?)"

Page 17: L9-14: I'm unclear as to what was designated a "match" for these spacer queries. did nucleotide sequences have to 100% match? Were you just looking for sequences that clustered with the teg genes (etc) at a 70% amino acid level like you used to cluster. Methods could be clearer to me here.

P25, L13: "detrimental" feels like the wrong word to use here

I don't think Figure 9 is necessary

P27: How many replicates were used in the worm survival assays per strain? Just one?

Supplemental Figure 1 and throughout: Please make sure to label what all the error bars are. Many times this information is in the axis, but here (and maybe other places) it isn't.

I think it would also be good to step the reader through how you accounted for phylogenetic signals within the GWAS assays and how you controlled for the multiple testing problem. You do both of this (and as far as I'm concerned you do a good job of controlling for these issues), but I could see how someone reading this would lose the idea that you took the time to control for both these issues. A couple of plain sentences describing how you looked for multiple signals and compared this against TREEwas to minimize phylogeny would be great. Likewise, a couple of sentences saying that you took care of the multiple tests issue by using permutations and creating your own distributions would also help out for clarity here.

**Response to reviewers**

***Reviewer #1:***

*In this manuscript, the authors assess the genetic and genomic bases of P. aeruginosa virulence against C. elegans hosts. They test a panel of P. aeruginosa isolates (some clinical, some environmental) for host mortality and host fecundity. Then, they use genetic association and genetics methodology to determine the portions of the pathogen genome associated with virulence in C. elegans. Overall, their findings support several previously identified pathogen loci linked to virulence and they also find novel loci associated with virulence. Of particular interest are teg blocks that are associated with decreases in pathogen virulence.*

*Overall, the findings are a significant advance in host-pathogen interactions and the experimental work is quite thorough. However, I have several major comments about the manuscript writing and statistical analyses.*

**Response**:

We appreciate the reviewer’s opinion that our research is thorough and brings forward a significant advancement to the field of host-pathogen interactions. Our revisited manuscript and the responses below address the reviewer’s concerns.

*1. The introduction does not motivate the study effectively. The authors convey in the introduction that they "addressed the sources and genomic correlates of variability in the virulence of distinct P. aeruginosa strains towards C. elegans" (page 2 lines 11-12). It is not clear to me from the current introduction why variability, or the source of variability, in P. aeruginosa virulence towards C. elegans is worth pursuing. What do you hope to learn from this pursuit and why? The first several paragraphs of the introduction don't seem to address this question. Given that many different strains of P. aeruginosa likely interact with many different strains of C. elegans in nature (and your work explores on the pathogen side of this genetic variation...as one host genotype is primarily used throughout), I recommend an introduction that is more focused on variability in P. aeruginosa. I believe this will be more effective at framing the idea that bacterial immune systems may play a role in moderating virulence.*

**Response**:

We have modified the introduction (page 2, L23-29) to better present the hypothesis behind our pursuit of studying bacterial-driven variability in virulence. In brief, we postulate that bacteria-driven variability in virulence reflects bacterial adaptation to distinct environments, where invertebrate-driven predation and host(s) identity and prevalence are variable niche features.

The analysis of host-driven variability in virulence would complement our present study but it is a separate endeavor that would rely on different premises. At present, we know (pmID: 19150845) that *P. aeruginosa* PA14 virulence is equal for two highly divergent *C. elegans* strains, under the same assay condition we have used in our study (i.e. full lawns that void worm behavioral differences). However, further investigations would be needed to better estimate the extent of host-driven variability in virulence.

*2. Host-pathogen interactions can vary broadly under different environmental conditions (see Leung, J. M., S. A. Budischak, H. Chung The, C. Hansen, R. Bowcutt, R. Neill, M. Shellman, P. n. Loke, and A. L. Graham. 2018. Rapid environmental effects on gut nematode susceptibility in rewilded mice. PLOS Biology 16:e2004108). This should be taken into account when assessing the loci underlying virulence in the lab. This does not diminish your results in any way, but it is important to recognize that virulence may be environmentally mediated and thus the loci underlying virulence may be conditional.*

**Response**: We fully agree with the reviewer’s remark that the environmental conditions can influence pathogenic relationships and the particular genes involved in them. Thus, the set of natural conditions under which the pathogenic relationships take place would be the most relevant to understand the evolutionary forces that shaped the *P. aeruginosa* genome. Among three experimental lab models for the interaction between *P. aeruginosa* and *C. elegans* (pmIDs: 9989496, 9892699, 23601103), the one we have chosen is the one where virulence depends on biofilm formation, the quorum sensing system, and host colonization by bacteria. Thus, our selected lab system approximates a naturally occurring mode of *P. aeruginosa* growth (*i.e*. biofilm).

Environmental effects that are the result of interactions between gut microbiota and the host, like the ones unveiled in the mentioned article by Leung JM et.al. are interesting but lie beyond our current scope. We present a univariate analysis, where the host-bacterial interactions are isolated into pairs (composed of the host and a given bacteria isolate), as a simple approach to understand their relationship in the absence of potential confounding variables (i.e. additional interacting bacterial strains or species). To touch on the above discussed points, we modified the manuscript passage on the experimental mode used for our virulence assays (page 4, L20-22).

*3. It does not seem statistically appropriate to use Spearman rank correlations on data sets in which some data categories have very few data points. For example, Supplemental Figure 6E has one Type IV R-M system with only a single data point. I recognize that this analysis and several others like it find no significant association between variables analyzed, so perhaps this is a moot point. However, it would seem more appropriate to exclude these small data sets from the analysis.*

**Response**:

We agree with the reviewer that claims of present/absent relationship between variables using small datasets should not be performed. Thus, we refrain from assessing statisitical relationship for variables with datasets containing less than 10 non-zero values. Thus, the spearman correlation is removed from supplemental figure 6D and 6E and the corresponding description in the text was amended (page 13, L28-29). This modification doesn’t affect any major claim of the manuscript.

*4. I generally understand the hypotheses the authors present in Figure 9, the model linking CRISPR and virulence. However, the model figure itself (Figure 9), does not make intuitive sense to me.*

**Response**:

Considering that both reviewer #1 and #3 have found issue with our graphical model (Figure 9), we have opted to remove it, also helping to shorten the manuscript length.

*5. Rather than ending the conclusion with potential missed loci, I recommend ending by expanding on the CRISPR and virulence association and what it might mean for bacterial genome evolution as a whole. Essentially put this result in a broader context.*

**Response**: We agree with the reviewer’s suggestion and we have reshuffled the discussion section. The section on the ‘potential missed loci’ is now presented earlier (page 16, L7-14). The discussion now concludes with our perspectives regarding the relationship between CRISPR-Cas systems and virulence, as suggested by the reviewer. Of note, we would not like to extrapolate our findings to a ‘one model fits all’ perspective, because not all host-pathogen interactions are driven by similar selection pressures, constraints and mechanisms.

***Reviewer #2:***

*This is a comparative genomic analysis of Pseudomonas aeruginosa isolates that differed in their virulence towards Caenorhabditis elegans. A number of accessory genes were identified that were associated with high-virulence or low-virulence isolates. Mutants containing disruptions of several of these genes and over-expression constructs were generated to measure the contribution of these gene to virulence towards C. elegans. Some of these showed the expected changes while others did not. Further analyses provided evidence supporting the model that active CRISPR-CAS systems are associated with high-virulence strains, and that some CRISP-CAS systems have targeted accessory genomic elements associated with low-virulence. The manuscript is in general well written and provides new information that the readership of Genome Biology will find interesting. There are, however, several issues that must be addressed before the strength of the conclusions can be assessed.*

**Response**:

We are thankful for the reviewer’s critique and its assessment of the paper as well written and of interest to the journal readership.

*Major points:*

*1. More information on the C. elegans virulence assays should be provided. In particular, approximately how many worms were tested for each isolate? Are the life-span values shown in Fig. 2 derived from a single plate of worms or from multiple plates? If multiple, how many?*

**Response**:

The requested experimental details of virulence assays have been described in more detail in the section ‘Worm survival assays (Virulence assays)’ of Material and Methods (page 18, L18-19). In brief, survival data summarizes a minimum of 3 plates with an average total n of 84 worms. Of note, our virulence measurement method (i.e. lifespan machine) has much higher temporal resolution than commonly used manual scoring method, leading to better estimates of median survival times. We also further verified our virulence measurements, that are in agreement with the traditionally used manual survival curve method (Supp. Figure 8.) and previously reported values for commonly used strains (e.g. PA14).

*2. It appears that no correction for multiple comparisons was made to the statistics describing the gene association studies. This should be done. It is anticipated that some of the HVA and LVA genes will no longer be statistically significantly associated with virulence phenotypes after this correction is made, and that this explains why several of the genes were thought to be associated with virulence but did not have a phenotype when deleted or over-expressed. If this is indeed the case, the discussion should be revised accordingly.*

**Response**:

We did control our results for multiple statistical testing using a permutation test approach (pmID: 18939983). To make this more explicit to the readers, the sentence covering the use of the permutation test in the result section has been modified (page 5, L20-25). Furthermore, the materials and methods section (page 22, L18-20 and page 22, L24-27) has also been modified to better explain the use of the permutation test to address multiple statistical testing. Thus, the reported gene associations are corrected for multiple hypothesis testing, and the absence of phenotypes for some of the tested genes is not due to false positives. Of note, we addressed multiple possible explanations for the presence of associated genes that do not have direct effects when deleted or over-expressed. This is found in the result section (page 9, L15-22) and in the discussion section (page 15, L29-30 and page 16 L1-6), where the possibility of statistical and phylogenetical spurious associations is evaluated, altogether with epistasis and the physical linkage of genes into ‘blocks’ as plausible biological explanations.

*3. LVA genes might also decrease virulence if their insertion in the chromosome disrupted a gene that enhanced virulence. For LVA genes that did not have the expected phenotypes, this possibility should be examined (i.e. their genomic context should be examined to see whether they appear to have inserted within genes).*

**Response**:

We have considered the possibility that LVA genes insert and disrupt HVA genes that the reviewer eludes to. Disruption of an HVA gene by the insertion of an LVA gene would lead to a truncated HVA gene. In our set, 28 of the 35 HVA genes (or 80% of the HVA genes) encode proteins with identical length in all isolates where the HVA gene is present. The remaining 7 HVA genes encode proteins with small amount of length variability between isolates. Analysis of the gene neighborhood for these 7 HVA genes, found no adjacent LVA genes. Altogether, we find no evidence for the proposal that LVA genes may be disrupting HVA genes. These negative results are not included in the manuscript, to avoid further increasing its length.

*4. The phylogenetic trees should be corrected to remove the effects of recombination regions to more accurately show the phylogenetic relationships between the isolates. In particular, TreeWas is meant to be used with trees for which recombination sequences have been removed.*

**Response**:

We applied the recombination-aware approach, ClonalFrameML (pmID: 25675341) to reconstruct the phylogenetic tree with corrected branch lengths, prior to using treeWas. There was a minor effect on the scores and the p-values reported by treeWas, however it did not affect the number of virulence associated genes that are supported by either the simultaneous or subsequent scores. We updated the methods section (page 21, L18-24) and the tables accordingly.

*5. page 19, lines 34-36: The reason stated for excluding type I-C CRISPR-Cas systems in this analysis is not compelling. One would expect that these ICE-encoded CRISPR-Cas systems would benefit the ICE by acting as standard immunity systems to prevent the death of the host bacterium and therefore should also prevent the uptake of LVA genes. Either a better rationale for excluding the type I-C systems should be provided or these systems should be included in the overall analysis.*

**Response**:

We have modified the paragraph (page 13, L1-3) to better explain our argument. We argue that ICEs encoded I-C CRISPR-Cas systems function **primarily** to the fitness benefit of the ICE itself and not as immune defense systems of the bacterial host.

Studied cases of MGE-encoded CRISPR-Cas systems have shown that such systems carry spacers that target: 1) host’s defense islands (pmID: 23446421); 2) other competing MGEs (pmID: 31165781). If the ICE’s I-C system targets other MGEs, it would provide a benefit to the host, as pointed by the reviewer. However, the extent to which such ICE provided benefit is comparable to the immunity endowed by host CRISPR-Cas I-E or I-F systems is unknown. Based on our results and the literature, we claim that such presumed benefit may be turn out to be negligible in several evolutionary timescales.

We first addressed CRISPR-Cas systems separated by subtypes and our analysis suggests that CRISPR-Cas I-C behave differently that CRISPR-Cas I-E and I-F. With the caveat of a small dataset due to the low I-C prevalence among isolates, we observe that strains with I-C systems have lower virulence than those without it (supplemental figure 5A, t-test, p = 0.03). Such association is contrary to the observations for strains with I-E or I-F systems, which associate with higher virulence (Figure 8C). Merging together CRISPR-Cas subtypes showing opposing associations with virulence would be inadequate.

Moreover, extensive screening efforts have unveiled an array of anti-CRISPR protein families that target I-E and I-F systems of *P. aeruginosa*. Remarkably, similar screens have found no anti- CRISPR targeting *P. aeruginosa* I-C system (pmID: 30190308). Such results, while negative, suggests that *P. aeruginosa* I-C systems do not exert significant immune selection against viruses to drive the evolution of inhibitory anti-IC genes, consistent with a minor or negligible role for I-C as bacterial immune system in the species.

*6. Supplemental Figure 5, panel D. Could the authors speculate on why the survival curves differ so*

*much between the PAO1 wildtype and the PAO1 with the empty plasmid?*

**Response**:

Plasmids induce fitness costs and can change bacterial gene expression (pmID: 30097663). In our case, it is plausible that the antibiotic and/or the antibiotic-resistance gene in the plasmid, induce a stress response in PAO1. Such response could modify the expression of some virulence factors. This proposed explanation is supported by findings in *P. aeruginosa,* where *rpoS*-mediated stress responses modify expression of virulence genes (e.g. pmID:10383954).

*7. Generation of z8 strains: Either more details should be provided or a reference should be added to help the reader who is unfamiliar with this strategy.*

**Response**:

The methodology to use the endogenous I-F CRISPR-Cas system of PA14 for gene editing has been documented in further detail in the methods section (page 19 L19-28 and page 20, L1-3). An example of the plasmid construct used for editing is now included in the supplemental material (Table 9).

*Minor points:*

*1. The Figure 3 panels are labeled incorrectly according to the Fig. 3 legend.*

*2. It is appreciated that repeatedly showing the "Median survival vs. Strain" curve helps the reader interpret the gene matrices in the different figures. However, since the repetition of this curve is shown to inform the axes of these matrices and not to provide new information, it should be included in the matrix figure panels and not shown as separate panels. For example, in figure 7 panel A and panel B should be combined into a single panel. Panel A provides no new information and should therefore not be a separate panel.*

*3. page 6, line 53: Should "mean" be "median"?*

*4. page 21, line 32: A parenthesis is missing after "Figure 7."*

**Response**:

All minor points were corrected as suggested by the reviewer. Figures 3 and 7 have been modified and references to them amended.

***Reviewer #3****:*

*The manuscript from Vasquez-Rifo et al. describes the use of a GWAS approach to identify genes of Pseudomonas aeruginosa that are correlated with higher and lower virulence in a slow killing model with C. elegans. Overall, there is quite a bit of work that went into this manuscript, and the results are solid.*

**Response**:

We appreciate the reviewer’s acknowledgment of the manuscript as interesting and with a good strength of evidence.

*My one main critique is that this manuscript is quite dense to read. I wonder if there is a way to parse the information more clearly and to *just* focus on the main results within the main body of the manuscript. As such there are a variety of tests and associations and it's a significant effort to wade through everything to digest the results that are worth digesting. I'm a fan of including as much information as possible, usually, but there is a lot in this manuscript and I think it really help the readers to move the negative results (other immune systems, some of the low virulence genes, etc...) to a supplemental document.*

**Response**:

We acknowledge that our manuscript is of considerable length, mostly due to the comprehensive view we aim to present. Following the reviewer’s advice, we have relocated and removed text that is not required to convey main results to the readers. The determination of CRISPRCas activity status based on self-targeting spacers has been relocated to the methods section (page 23, L26-30 and page 24, L1-2). The paragraphs describing the RM systems and novel defense systems are now shortened by removal of descriptive sentences that aren’t strictly necessary to convey main results (page 13, L26 and page 14, L2). In addition, figure 9 has now been removed from the manuscript.

*Other than this main critique on clarity, I really only have a few minor points that the authors should consider before publication:*

*Page 2, L31 and throughout: you keep using the word "indirect" here. I think it would be good, at least once, to mention that these are truly correlations and that doesn't imply causation. Using the word "indirect" kind of glosses over this point, the association is real, it's just not necessarily a causative association.*

**Response**:

We have modified the passage in question to enhance clarity (page 3, L9). We use the term ‘indirect’ to refer to effects that are mediated by an additional component or variable that has a ‘direct’ effect (i.e. an effect on virulence that is measurable by the genetic testing we conducted). In particular, we propose an ‘indirect’ role for the CRISPR-Cas system in shaping bacterial virulence (page 3, L12- 15), because the system functions to filter out MGEs that are ‘directly’ acting to decrease virulence, such as the *teg* block. This proposal is based on the correlative evidence provided AND the genetic testing conducted, where the MGE studied (i.e. teg block, a CRISPR-Cas restricted element) affects virulence, while CRISPR-Cas does not. To refer to statistical based association, we use the terminology ‘association’ or ‘correlation’.

*Page 2, Line 58 (and a couple of other places, page 3 L6 etc...): there are statements included like "and do not associate with any clade in particular". Use of this statement is inherently subjective. It may look like there are no associations to the eye, but there might be some there still. Would be good to include some data showing lack of bias in the sampling of strains or the phylogenetic clustering of strains to back up statements like this.*

**Response**: We agree with the reviewer and have modified the statements in (page 4, L5-6 and L12).

*Figure 3: I think the labels are off here as the legend specifies A-D but the labels go from A-E. Page 13, L29-33: I think there's a typo here. I think the second use of qsrO should actually read ghlO?..."In contrast, no difference in virulence was observed for strains overexpressing the qsrO (?)"*

**Response**: The legend of figure 3 has been fixed. The ‘*qsrO*’ typo (now on page 9, L6) is fixed too.

*Page 17: L9-14: I'm unclear as to what was designated a "match" for these spacer queries. did nucleotide sequences have to 100% match? Were you just looking for sequences that clustered with the teg genes (etc) at a 70% amino acid level like you used to cluster. Methods could be clearer to me here.*

**Response**:

The method section (page 24, L8-12) has been amended to better explain the criteria used to determine the target of spacers. In brief we used blast to identify match between spacer sequences and genes. Blast hits in which the alignment started after position 2 in the spacer query or had less than 95% identity were filtered out. The gene clusters containing the remaining hits were considered as CRISPR targets.

*P25, L13: "detrimental" feels like the wrong word to use here*

*I don't think Figure 9 is necessary*

**Response**:

The word ‘detrimental’ has been replaced by ‘decrease’ (page 16, L15). Figure 9 has been removed.

*P27: How many replicates were used in the worm survival assays per strain? Just one?*

**Response**:

The requested experimental details of virulence assays have been described in more detail in the section ‘Worm survival assays (Virulence assays)’ of Material and Methods (page 18, L18-19). In brief, survival data summarizes a minimum of 3 plates (independent worm populations) with an average total n of 84 worms.

*Supplemental Figure 1 and throughout: Please make sure to label what all the error bars are. Many times this information is in the axis, but here (and maybe other places) it isn't.*

**Response**:

All figures legends have been revisited to ensure they include all details of the plotted data. The lines in the Suppl. Figure 1 corresponds to the whiskers of the box plots. To make more explicit this standard graph representation the ‘Box plot’ term is now referred to as ‘Box-and-whisker’ plot.

*I think it would also be good to step the reader through how you accounted for phylogenetic signals within the GWAS assays and how you controlled for the multiple testing problem. You do both of this (and as far as I'm concerned you do a good job of controlling for these issues), but I could see how someone reading this would lose the idea that you took the time to control for both these issues. A couple of plain sentences describing how you looked for multiple signals and compared this against TREEwas to minimize phylogeny would be great. Likewise, a couple of sentences saying that you took care of the multiple tests issue by using permutations and creating your own distributions would also help out for clarity here.*

**Response**:

Following the reviewer’s advice, we have modified the text passages on the use of the treeWAS method (page 5, L24-27). Also, the sentences explaining the use of the permutation test have been modified in the result section (page 5, L22-24) and the materials and methods section (page 22, L2-17).

**2nd round**

**Reviewer 1**

Overall, the authors have addressed the concerns I raised in my previous review. I believe the manuscript is significantly improved by their responses to each of the reviews.

I have one major comment regarding the revised introduction. While I appreciate that the authors now provide a more substantial framework for motivating this work, I believe it is likely not justified to infer an evolutionary history between C. elegans and P. aeruginosa. At least from the information presented in the introduction, there seems to be little to no evidence that these species share an evolutionary history. The authors either need to provide clear documentation of this evolutionary history, or find another means of motivating their study. Importantly, there is no need to invoke a very specific relationship between C. elegans and P. aeruginosa. Rather, the authors could take a broader approach, invoking P. aeruginosa interactions with a potentially broad range of hosts in nature (or through evolutionary time), of which C. elegans may be representative.

**Reviewer 2**

The authors have carefully addressed the prior criticisms.

**Response to Reviewers**

***Reviewer 1:***

*We have modified our manuscript to comply with all the formatting requirements mentioned in the decision letter. Also, in order to address the remaining critique of reviewer #1, we have edited the introduction to avoid the interpretation that C.elegans and P.aeruginosa have coevolved. In particular, we modified or added the sentences shown below:*

*page 2, L14: "This mutually-antagonistic relationship between C. elegans and P. aeruginosa is a well-studied model for ecologically coexisting predators of P. aeruginosa that are also natural hosts for infection [8]." now reads: "This mutually-antagonistic relationship between C. elegans and P. aeruginosa is a well-studied model that serves as a proxy for ecologically coexisting predators of P. aeruginosa that are also natural hosts for infection [8]."*

*page 2, L21 added sentence:*

*"Nonetheless, independently of their putative co-existence in the wild, C. elegans can be used as a experimentally tractable proxy of naturally occuring predator and host of P. aeruginosa."*
